# Concomitant Kinase-Dead BRAF and Oncogenic KRAS Lead to an Aggressive Biologic Behavior and Tumor Lysis Syndrome: A Case Report

**DOI:** 10.3389/fonc.2022.885814

**Published:** 2022-05-02

**Authors:** Roy Holland, Offir Ben-Ishay, Irit Ben-Aharon

**Affiliations:** ^1^ Division of Oncology, Rambam Health Care Campus, Haifa, Israel; ^2^ Division of Surgery, Rambam Health Care Campus, Haifa, Israel; ^3^ Rappaport Faculty of Medicine, Technion, Haifa, Israel; ^4^ Technion-Integrated Cancer Center, Technion, Haifa, Israel

**Keywords:** tumor lysis syndrome, case report, concomitant BRAF and KRAS mutations, duodenal mucinous adenocarcinoma, NGS - next generation sequencing

## Abstract

Tumor lysis syndrome (TLS) is a life-threatening oncological emergency rarely seen in solid tumors and is a complication of cancer therapy for rapidly proliferating tumors with devastating outcomes. BRAF and KRAS are two key oncogenes in the MAPK signaling pathway that are routinely examined for mutations to predict resistance to anti-EGFR therapy. Concomitant KRAS and BRAF mutations in GI tumors are rare, occurring in less than 0.001% of cases and are associated with an aggressive tumor behavior. We report an unusual case of a young male patient diagnosed with locally advanced duodenal mucinous adenocarcinoma harboring concomitant KRAS and BRAF mutations. This unique genetic profile generated hyperactivation of the EGFR signaling pathway. Following day-1 of mFOLFOX-6 chemotherapy protocol, the patient developed TLS. Clinical resolution was achieved using high volume hydration. Unfortunately, the patient passed away 10 days later during anesthesia induction.

## Introduction

Small bowel cancer (SBC) is rare and accounts for only about 0.6% of total cancer cases and about 3.3% of all gastrointestinal (GI) tumors (1, 2). Despite their rarity, neoplasms of the small bowel are on the rise, with an estimated growth of more than 100% in the incidence rate over the past four decades (3). Within the small bowel, 50% of tumors are found in the duodenum. There are more than 45 different histological types of SBC; Most frequent are neuroendocrine tumors (NETs) which account for 37.4% of the SBCs followed by small bowel adenocarcinoma (SBA) (4). The rarity of SBCs and the fact that most patients present with nonspecific symptoms, make the diagnosis challenging, with some reports estimate 12 months from symptoms onset to diagnosis.

Tumor Lysis Syndrome (TLS) is an oncological emergency caused by massive tumor cell destruction leading to a release of cellular content into the blood stream. The Cairo-Bishop definition provides specific criteria for the diagnosis (**Table 1**), which is characterized by laboratory findings of hyperuricemia, hyperkalemia, hyperphosphatemia, and hypocalcemia (5).

**Table 1:** Laboratory TLS (LTLS) is defined for any two or more serum values described above.

Uric Acid	≥ 8 mg/dL or 25% increase from base line
Potassium	≥ 6 mEq/L or 25% increase from base line
Phosphorus	≥ 6.5 mg/dL or 25% increase from base line
Calcium	≤ 7 mg/dL or 25% decrease from base line

A clinical TLS (CTLS) is defined as the presence of LTLS and any one or more of the following conditions: (A) An elevated creatinine level of more than 1.5 times the upper limit of normal (ULN) adjusted to age and gender. (B) Cardiac arrhythmia or sudden death. (C) Seizure.

Acute renal failure and a rising creatinine level are secondary to acute uric acid crystal nephropathy and calcium phosphate deposition. TLS most often develops in patients with high grade hematological malignancies, in particular Burkitt lymphomas and acute lymphoblastic leukemia (ALL), shortly after initiation of chemotherapy. TLS has been rarely described in the setting of solid tumors (6). Only 17 cases of TLS in liver cancer,10 in colon cancer, 10 in gastric cancer and none in small bowel cancer had been reported thus far (7).

Herein, we describe a young patient who was diagnosed with primary mucinous adenocarcinoma located in the third part of the duodenum. The patient presented with hypercalcemia, received one partial course of mFOLFOX-6 chemotherapy protocol which was discontinued due to a severe tumor lysis syndrome. The patient later suffered a cardiac arrest and died.

## Case Presentation

### Diagnosis

**Figure 1:**
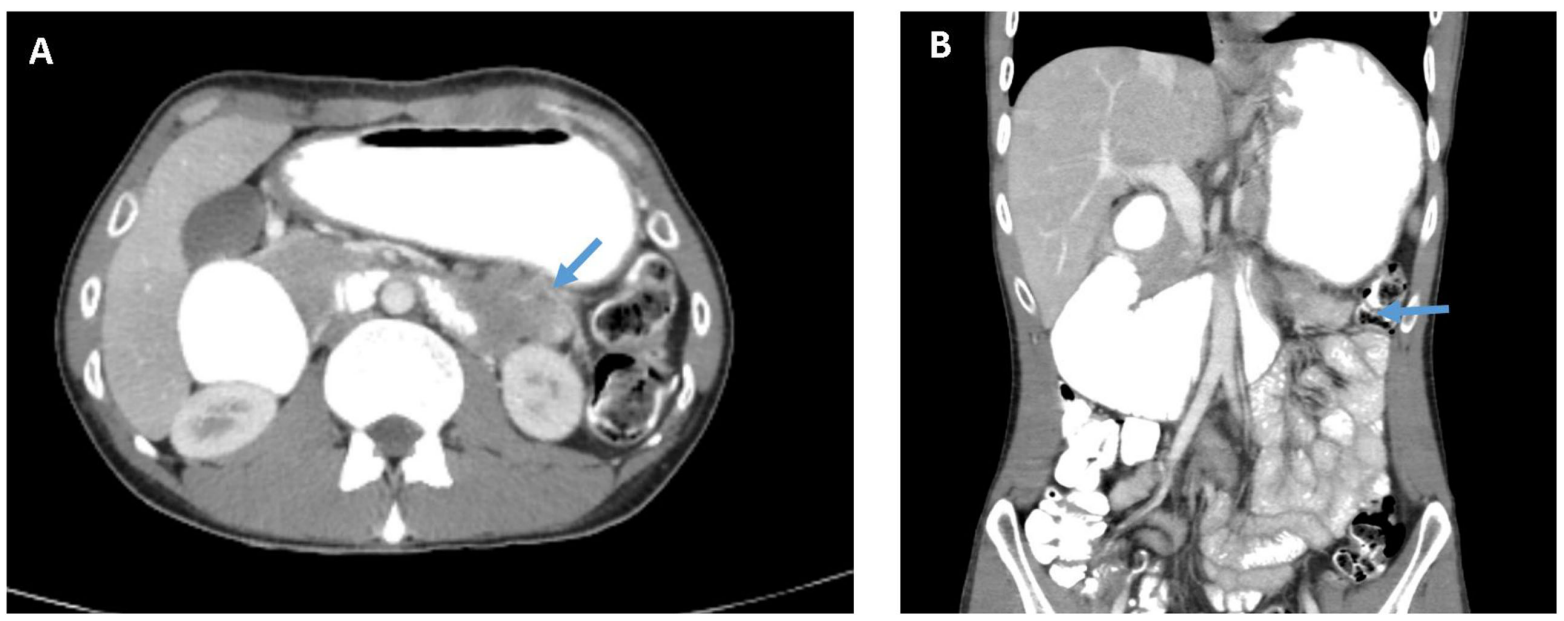
CT of the abdomen and pelvis with intravenous and oral contrast material. **(A)** Axial view showing the tumor in the 4^th^ part of the duodenum (see arrow) involving the hilum of the left kidney. **(B)** Coronal view showing evident small bowel obstruction and gastric outlet obstruction.

A 25-year-old male patient with no significant medical history presented with early satiety, bloating, epigastric discomfort, and intermittent vomiting over a month. He was referred to a community hospital, underwent a gastroscopy, diagnosed with H.Pylori gastritis and started standard eradication treatment. A week later the patient was admitted to a tertiary hospital (RHCC – Rambam Health Care Campus) with persistent complaints of vomiting and inability to eat. The underlying cause was unknown. Routine blood tests showed no abnormality. A computed tomography (CT) of the abdomen and pelvis (**Figure 1**) revealed a large mass (5.3x3.9cm) with extensive regional lymphadenopathy deriving from the fourth part of the duodenum. The mass invaded the hilum of the left kidney with no evident dissection plane from the tail of the pancreas. The tumor caused small bowel obstruction with prominent dilation of the proximal duodenum and clinically evident gastric outlet obstruction. Distant metastases were not shown on chest nor abdominal CT scans.

**Figure 2:**
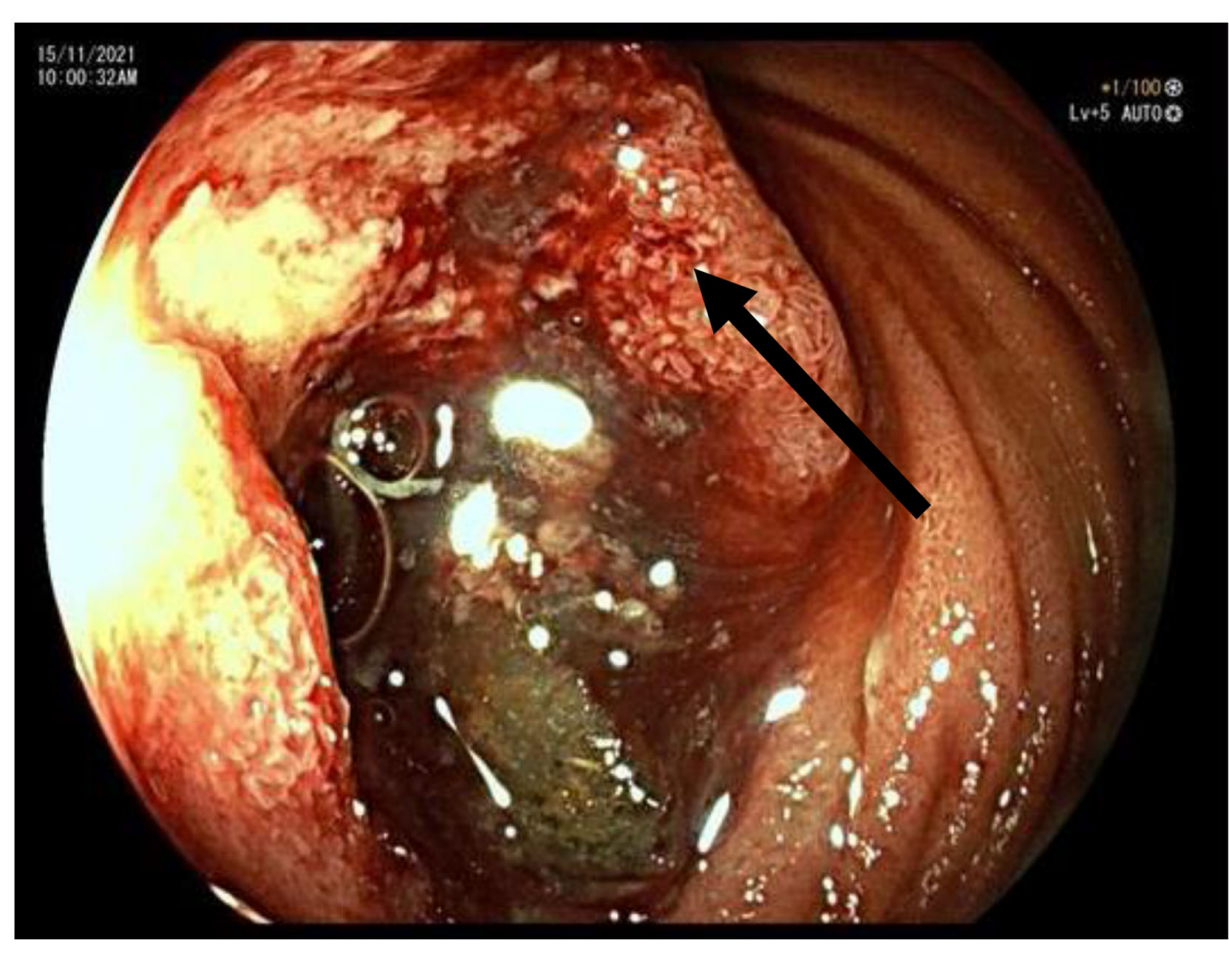
Endoscopic image of the tumor.

**Figure 3:**
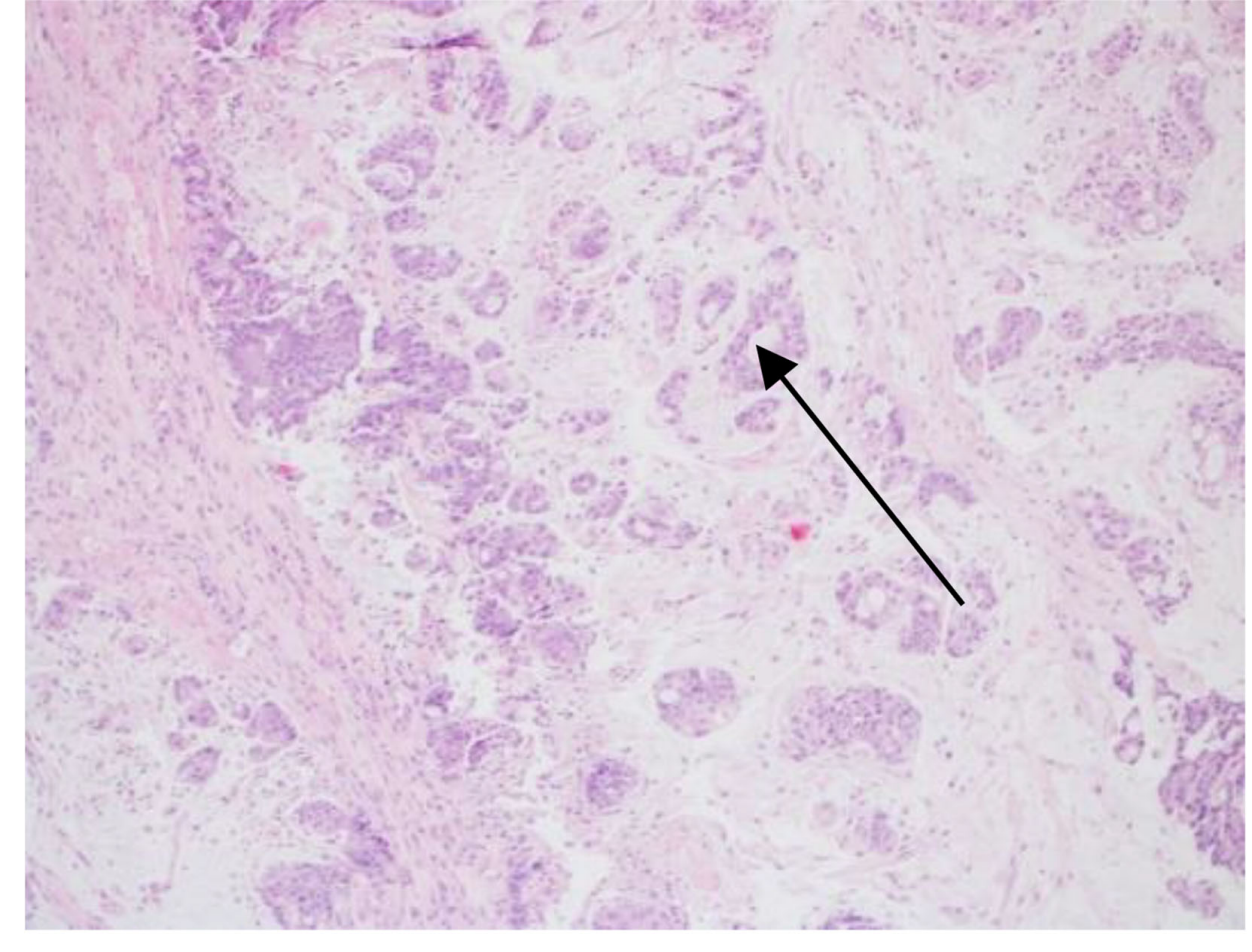
H&E stain X100. Arrow points mucin producing malignant cells.

Second endoscopy was performed, which identified an intralumenal large mass between the third and fourth part of the duodenum (**Figure 2**). Biopsies were taken and histopathology examination disclosed mucinous adenocarcinoma of the duodenum (**Figure 3**). Tumor was locally advanced, adjacent to the pancreas and renal vasculature and was deemed borderline resectable. Multidisciplinary team advised neoadjuvant approach due to expected extent of the surgical resection of an infiltrating large tumor. Modified FOLFOX-6 (oxaliplatin 85 mg/m^2^ and leucovorin 400 mg/m^2^ as a 2-hour infusion, followed by 5-fluorouracil 400 mg/m^2^ as a bolus and 2,400 mg/m^2^ as a 46-hour infusion) was selected as a neoadjuvant protocol.

### Hypercalcemia of Malignancy

Prior to the neoadjuvant treatment commencement, blood work revealed calcium levels of 14.4 mg/dL (normal range 8.5-10.2 mg/dL) most probably due to paraneoplastic section of PTHrP, as no skeletal lesions were documented on imaging. Primary hyperparathyroidism was excluded as PTH levels were diminished. Management of hypercalcemia with hydration and calcitonin yielded normalization of calcium levels.

### Tumor Lysis Syndrome

Following day-1 of protocol (Oxaliplatin, leucovorin and bolus 5- fluorouracil) and 24 hours of continuous 5-FU, the patient became anuric with altered mental status. Chemistry panel indicated creatinine level of 5.42 mg/dL (baseline two days before was 0.72 mg/dL and normal range of 0.7-1.3 mg/dL), phosphate level of 11.51 mg/dL (Normal range 2.3-4.7 mg/dL) and uric acid level was 14.62 mg/dL (Normal range 3.5-7.5 mg/dL), thus, a diagnosis of CTLS was established. Chemotherapy infusion was discontinued immediately, and high volume of normal saline hydration was initiated. The patient neurological condition was improving and blood tests were normalized again.

### Surgical Intervention

**Figure 4:**
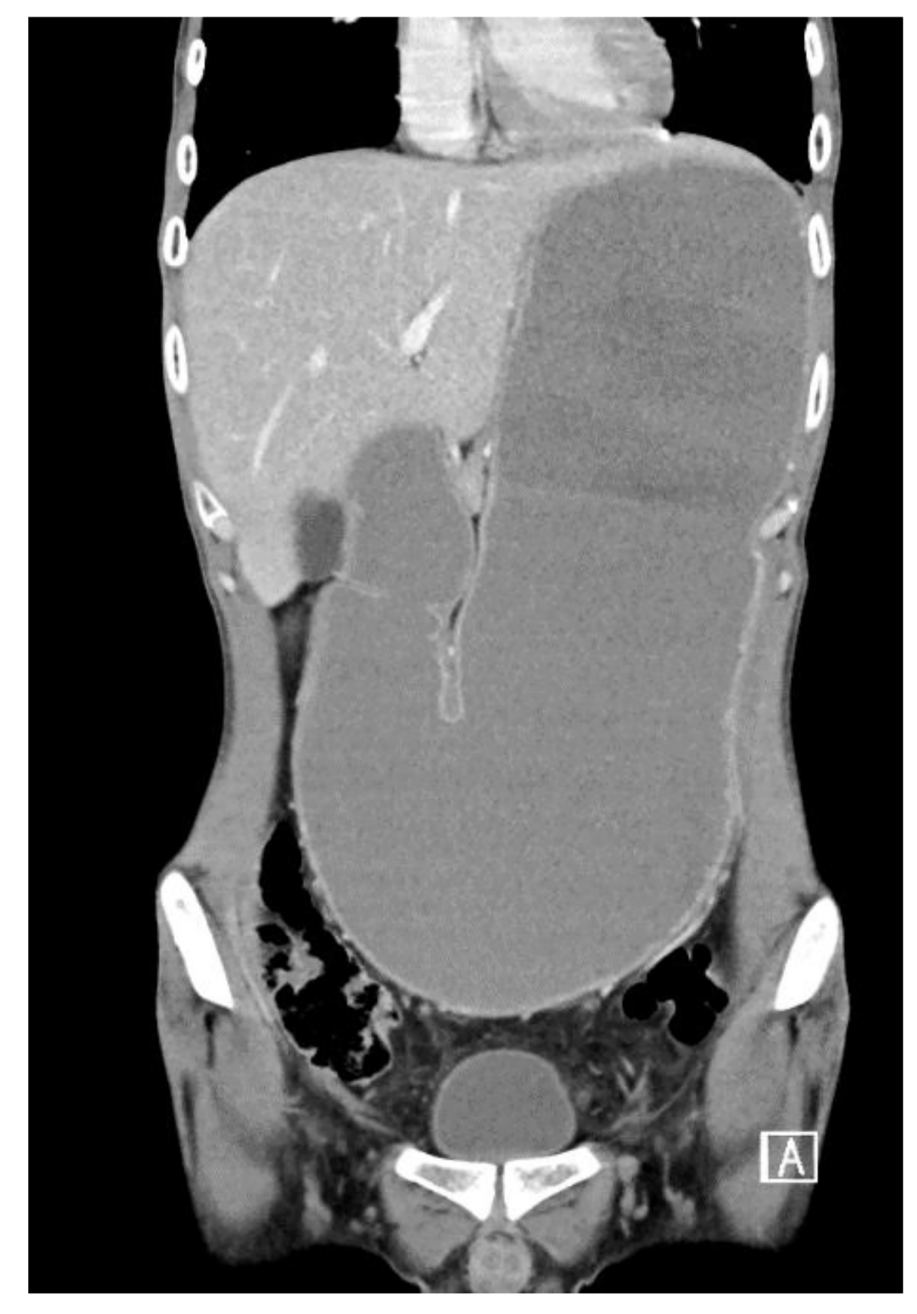
Restaging CT.

Due to the life-threatening TLS, our GI multidisciplinary team decided to withhold the neoadjuvant treatment and proceed to surgical resection of the lesion. A pre-op restaging CT scan was performed without enteral contrast agent due to duodenal obstruction. The CT demonstrated extreme gastric dilation, with no significant change in the tumor location and size (**Figure 4**).

The patient was taken to the operation room after 10 days of proper nutritional support with total parenteral nutrition, electrolytes balance and fluid supplementation, while maintained on nil per Os. During anesthesia induction the patient suffered a sudden cardiac arrest and resuscitation efforts unfortunately failed.

### Mutational Analysis

The biopsy specimen was sequenced using next-generation sequencing (NGS) performed on genomic DNA isolated from a micro-dissected, formalin-fixed paraffin-embedded tumor sample using the illumine NovaSeq 6000 sequencers. The results revealed a mutated BRAF (exon 15, p.D594N, c.1780G>A) as well as a mutated KRAS (exon 3, p.A59E, c.176C>A). Microsatellite instability (MSI) stable and tumor mutational burden (TMB) of 10, mutations in MED12 (exon 2, p.G44S, c130G>A), PIKCA (exon 3, p.G118D, c.35G>A) and TP53 (exon 5, p.P152L, c.455C>T).

## Discussion

This is a case report of an exceptional presentation of duodenal carcinoma in a very young patient manifested with severe TLS. The clinical features of this case are unusual by age of onset as well as tumor location and histology. Primary duodenal tumors are rare, median age of onset is at the seventh decade, 50-75% of duodenal cancers are located in the 2^nd^ part of the duodenal with only about 15% located in the third part. Mucinous adenocarcinoma is also considered extremely infrequent histological feature of duodenal cancer. Furthermore, the tumor genetic landscape of concomitant BRAF and KRAS mutation is an uncommon finding. Lastly, TLS is not frequently seen in solid tumors, in particular, GI tract carcinomas.

The MAP-kinase signaling pathway plays a pivotal role in cell proliferation. The pathway composed of an extracellular receptor for epidermal growth factor that activates the RAS-RAF-MEK-ERK chain. BRAF and KRAS are two key oncogenes in the MAP-kinase signaling pathway. The genomic alterations in SBA somewhat resemble the ones found in colorectal cancer (CRC). BRAF and KRAS mutations occur at similar frequencies in both SBAs and CRCs, 10% and 50% respectively (8, 9). Codon 12 (G12D mutation) is the most frequent hotspot in KRAS mutated CRCs and SBAs. An interesting difference can be found in the pathological variant of BRAF mutated cancers. V600E mutations are found in 73% of BRAF mutated CRCs with only less than 10% of the BRAF mutated SBAs (8, 10). While previously considered to be mutually exclusive, concomitant mutations in both BRAF and KRAS have been reported (11) with an occurrence of less than 0.001% in GI tumors (12). In the case reported here, not only both BRAF and KRAS were mutated, but harbored a rare combination of variants. BRAF^D594N^ is a class 3 kinase-dead mutation that in contrast to activation BRAF mutants, such as BRAF^V600E^, does not decrease the RAS-GTP activity (13). Hence, in this case, a Kinase-dead BRAF in the presence of an oncogenic KRAS cooperate to drive tumor progression *via* hyperactivation of CRAF and consequently elevated ERK signaling (14). The Kinase-dead BRAF^D594N^ is not autoinhibited and is recruited to the plasma membrane by RAS, where it binds to CRAF and acts as a scaffold to enhance CRAF activity and consequently enhance signaling through the pathway. This molecular mechanism suggests a more aggressive disease course and poorer outcome in patients with these coexisting mutation tumors (15, 16).

In summary, TLS is a rare side effect of chemotherapy for solid tumors. Few reports described TLS in GI solid tumors. This case is the first to report TLS in a Small Bowel Adenocarcinoma. There might be a correlation between the genetic aberrations of this case and the aggressive nature of the tumor and TLS. We speculate that hyperactivation of the MAPK signaling pathway led to an increased metabolic activity in the tumor cells and therefore the introduction of chemotherapy agents resulted in their rapid and massive destruction.

## Data Availability Statement

The original contributions presented in the study are included in the article/supplementary material. Further inquiries can be directed to the corresponding author.

## Author Contributions

RH and OB-I wrote the manuscript. IB-A supervision. All authors contributed to the article and approved the submitted version.

## Conflict of Interest

The authors declare that the research was conducted in the absence of any commercial or financial relationships that could be construed as a potential conflict of interest.

## Publisher’s Note

All claims expressed in this article are solely those of the authors and do not necessarily represent those of their affiliated organizations, or those of the publisher, the editors and the reviewers. Any product that may be evaluated in this article, or claim that may be made by its manufacturer, is not guaranteed or endorsed by the publisher.
